# Cell-Based Luciferase Assay for Testing SARS-CoV-2 3CL Protease Inhibitors

**DOI:** 10.3390/bios16050253

**Published:** 2026-04-30

**Authors:** Dmitry N. Shcherbakov, Ekaterina D. Mordvinova, Vadim O. Trufanov, Natalia V. Volkova, Yulia V. Meshkova, Maria K. Marenina, Anna V. Zaykovskaya, Ekaterina A. Volosnikova, Sophia S. Borisevich, Svetlana V. Belenkaya

**Affiliations:** 1 State Research Center of Virology and Biotechnology VECTOR, Rospotrebnadzor, 630559 Novosibirsk, Russia; scherbakov_dn@vector.nsc.ru (D.N.S.); mordvinova97@mail.ru (E.D.M.); trufano8@mail.ru (V.O.T.); tasha_wolkowa11.93@mail.ru (N.V.V.); zaykovskaya_av@vector.nsc.ru (A.V.Z.); volosnikova_ea@vector.nsc.ru (E.A.V.); 2 Research Institute of Biological Medicine Center for Recombinant Technologies, Altay State University, 656049 Barnaul, Russia; 3 N. N. Vorozhtsov Novosibirsk Institute of Organic Chemistry, Siberian Branch of the Russian Academy of Sciences, Academician Lavrent’ev Ave. 9, 630090 Novosibirsk, Russia; meshkova@nioch.nsc.ru (Y.V.M.); mareninamk@nioch.nsc.ru (M.K.M.); 4 Synchrotron Radiation Facility, Siberian Circular Photon Source “SKlF” Boreskov Institute Catalysis, Siberian Branch of the Russian Academy of Sciences, Nikolskiy pr-t, 1, Koltsovo, 630559 Novosibirsk, Russia; monrel@mail.ru

**Keywords:** SARS-CoV-2, 3CLpro, high-throughput screening, viral proteases, luciferase, antiviral inhibitor

## Abstract

A cell-based screening system for viral protease inhibitors was developed using firefly luciferase fragment complementation and validated on the SARS-CoV-2 3CLpro model. The optimal luciferase variant incorporating the VLQSGF proteolytic site (Luc III) retained 88% of its native activity. A critical requirement for system performance was the use of an extended nsp4–nsp6 fragment of the viral polyprotein rather than the mature protease, underscoring the importance of the native context for 3CLpro activity. The bicistronic construct pCAG-Luc-III-IRES-nsp4-6 enables coordinated expression of the reporter and protease, thereby increasing assay reproducibility. IC_50_ values obtained in this system for nirmatrelvir and GC376 correlated with live-virus assay data but differed significantly from those of a cell-free FRET assay, reflecting the impact of cellular barriers. This approach combines simplicity, a standard substrate, and high reproducibility, making it promising for high-throughput screening in basic laboratory settings and adaptable to other viral proteases.

## 1. Introduction

Virus-specific proteases play a key role in the viral replication cycle, which makes them attractive targets for the development of antiviral agents [1,2]. The interest in proteases as therapeutic targets is driven by several factors. First, they have a relatively simple structure compared with other viral proteins (such as polymerases or surface glycoproteins), which makes the production of their recombinant forms a routine task [3]. Second, their activity can be readily detected in vitro through cleavage of peptide bonds within either recombinant proteins or synthetic peptides.

The most common approach for the high-throughput screening of potential inhibitors involves the use of peptide substrates based on fluorescence resonance energy transfer (FRET) [4,5]. This method is characterized by its speed, high sensitivity, and simplicity (or “ease of use”). However, the results obtained with this method do not always correlate with data from in vivo antiviral assays or cell culture tests [6].

An alternative to cell-free systems is provided by cell-based assays. In such systems, the synthesis of viral proteases occurs under conditions that closely mimic the native environment [7,8], and the tested compounds are exposed to an intracellular environment, enabling simultaneous assessment of their inhibitory activity and cytotoxicity. However, detecting proteolysis within cells requires the use of specialized reporter systems. Optical methods employing reporter genes (luciferase, chloramphenicol acetyltransferase, β-galactosidase, fluorescent proteins, etc.) hold a leading position in this field. Among these, luciferase-based assays offer the highest sensitivity and broadest dynamic range [9].

Among the multitude of described luciferases (Firefly (Luc), Renilla (RLuc), NanoLuc, etc.) [10], the firefly luciferase from *Photinus pyralis* deserves special attention. This enzyme (Luc) catalyzes a reaction involving D-luciferin, ATP, Mg^2+^, and oxygen, which is accompanied by highly efficient light emission in the visible spectrum (540–600 nm) with a quantum yield of 0.88 [11,12]. Thanks to its high signal-to-noise ratio, low substrate toxicity, and compatibility with various formats (lysed and live cells), Luc has become one of the most sought-after reporter systems for developing intracellular biosensors [13]. To date, a number of methodological approaches have been developed for creating Luc-based biosensors: split Luc systems, bioluminescence resonance energy transfer, circularly permuted Luc, circular Luc, and dual Luc [14].

These approaches have also been applied to study viral proteases and screen libraries of inhibitor compounds. A biosensor based on circular Luc was developed for the rapid assessment of hepatitis A virus cysteine protease activity in HEK293T cells [15,16]. A similar system was described for both papain-like protease and 3C-like protease (3CLpro) of MERS-CoV and SARS-CoV-2 [17,18]. Recently, a system based on complementation of the highly sensitive NanoLuc luciferase was described for analyzing SARS-CoV-2 3CL protease [19]. However, existing Luc sensors for SARS-CoV-2 3CLpro [17] either employ less common modifications (circular permutation) or, as in the case with NanoLuc [18], require specific and more expensive substrates. There remains a need for a simple, reproducible, and cost-effective cell-based system based on classical Luc that combines reliable detection with the potential for straightforward adaptation to high-throughput screening in laboratories with basic equipment.

The objective of this study was to develop an adaptable cell-based system for testing viral protease inhibitors using firefly luciferase fragment complementation and to validate it on the SARS-CoV-2 3CL protease as a model to demonstrate its efficacy and specificity.

## 2. Materials and Methods

### 2.1. Cell Lines and Reagents

The HEK293T cell line was provided by the Department “Collections of Microorganisms” of the Rospotrebnadzor State Research Center Vector (Novosibirsk, Russia). Cells were cultured on Dulbecco’s Modified Eagle Medium (DMEM) (Invitrogen, Carlsbad, CA, USA) and Dulbecco’s Modified Eagle Medium/Nutrient Mixture F-12 (DMEM/F12) (SRC Vector, Novosibirsk, Russia), with the addition of 10% (*v*/*v*) fetal bovine serum (FBS) (Invitrogen, Carlsbad, CA, USA), and 0.6 mg/mL L-glutamine (Invitrogen, Carlsbad, CA, USA) and 50 µg/mL gentamicin.

### 2.2. Molecular Modeling

The tertiary structure of the luciferases, including the reference structure, was predicted using the ColabFold platform. The primary metric employed was the local distance difference test (LDDT), which assesses local distances between all atoms in the model, including stereochemical plausibility checks. In all cases, pLDDT values exceeded 90% for the majority of the protein structures.

### 2.3. Plasmid Vector Construction

Plasmid vectors containing the luciferase gene with an inserted sequence encoding the 3CLpro cleavage site (VLQSGF) were constructed via site-directed mutagenesis (Q5 Site-Directed Mutagenesis Kit, NEB, Ipswich, MA, USA) using the commercially available pCAG-luciferase plasmid (hereafter pCAG-Luc). Plasmids pCAG-Luc-I, pCAG-Luc-II, and pCAG-Luc-III were generated using the primer pairs Luc-I-F/Luc-I-R (5′-gtgctgcagagcggcttcgaggtgcctaaaggactgaccg-3′/5′-gtccacgaacacaacaccaccg-3′), Luc-II-F/Luc-II-R (5′-gtgctgcagagcggcttcgccaagaagctgcgcg-3′/5′-cttggcggttgtaacctggc-3′), and Luc-III-F/Luc-III-R (5′-gtgctgcagagcggcttcaaaaccatgaccgagaaggagatcg-3′/5′-accgtgttccagcacgacg-3′), respectively.

The nucleotide sequences of the SARS-CoV-2 genomic nsp4-nsp6 region and mature 3CL protease (hereafter s3CL) were amplified via RT-PCR from viral RNA using the BioMaster RT-PCR kit (Biolabmix Ltd., Novosibirsk, Russia) and primers nsp4-F/nsp6-R (5′-aaaaaaGAATTCgccaccatgggtggtaaaattgttaataattggttgaagc-3′/5′-aaaaaGCGGCCGCTTAacccttgattgttcttttcactgc-3′) and s3CL-F/s3CL-R (5′-aaaaaGAATTCagtggttttagaaaaatggcattccca-3′/5′-aaaaaGCGGCCGCTTAacccttgattgttcttttcactgc-3′), respectively. The nsp4-nsp6 and s3CL sequences were cloned into the pCAG vector at unique restriction sites (EcoRI/CciNI), yielding the recombinant vectors pCAG-nsp4-6 and pCAG-s3CL.

The bicistronic construct enabling co-expression of the Luc-III variant gene (containing the cleavage site insertion (VLQSGF)) and the SARS-CoV-2 coronavirus polyprotein fragment (nsp4-nsp6) in a single reading frame was assembled via Gibson assembly. For this purpose, the synthetic EMCV IRES sequence and the nsp4-nsp6 genomic fragment were amplified using primers IRES-F/IRES-R and Gnsp4-F/Gnsp6-R. The amplicons were ligated together with the pCAG-Luc-III vector backbone (digested with CciNI/Bse21I) using the Gibson Assembly Master Mix (NEB, Ipswich, MA, USA), resulting in the recombinant vector pCAG-Luc-III-IRES-nsp4-6.

### 2.4. Transfections and Luciferase Assays

HEK 293T cells were seeded into 96-well plates, using 1 × 10^4^ cells per well in 0.1 mL of culture medium. Cells were transfected 24 h later with 50 ng plasmid DNA, using Lipofectamine 3000 (Invitrogen, Carlsbad, CA, USA). To control transfection efficiency, parallel wells were transfected with the pCAG-GFP plasmid (50 ng/well) in each independent experiment. At 24 h post-transfection, GFP-positive cells were visualized using a fluorescence microscope Leica DMi8 (Leica Microsystems GmbH, Wetzlar, Germany), and the percentage of fluorescent cells was estimated in 5–10 fields per well using ImageJ software (Version 1.54i). Only experiments with transfection efficiency in the range of 70–85% (mean 78 ± 6%, *n* = 12) were included in the analysis. The compounds were added 1 h post-transfection to final concentrations ranging from 0.1 to 100 µM. Luciferase activity was measured 30 h post-transfection. The growth medium was removed from the cells, and 30 μL of lysis buffer (Promega, Madison, WI, USA) was added. Next, a 25 μL aliquot of the lysate was transferred into a black optical plate, and the luminescence signal was measured using a Varioskan LUX instrument (Thermo Fisher Scientific, Waltham, MA, USA) with automatic injection of the luciferase substrate (35 μL/well).

### 2.5. Evaluation of Inhibitor Activity in the Developed Cell-Based System

HEK293T cells were seeded into 96-well plates at 1 × 10^4^ cells per well in 100 μL of culture medium. After 24 h, cells were transfected with plasmid DNA (50 ng/well) using Lipofectamine 3000 (Invitrogen, Carlsbad, CA, USA). For studies with the inhibitor nirmatrelvir (LEAPChem, Hangzhou, China), it was added at 1 h post-transfection across a range of final concentrations from 0.04 to 88.7 μM.

At 30 h post-transfection, the culture medium was removed, and 30 μL of lysis buffer (Promega, Madison, WI, USA) was added. A 25 μL aliquot of the lysate was transferred to a 96-well plate. The Varioskan LUX instrument (Thermo Fisher Scientific, Waltham, MA, USA) was used in automatic injection mode to add the luciferin-containing substrate (100 μL/well, Luciferase Assay System (Promega, Madison, WI, USA)), and luminescence was measured.

For Z′-factor determination, HEK293T cells were seeded in 96-well plates (1 × 10^4^ cells/well) and transfected with 50 ng/well of pCAG-Luc-III-IRES-nsp4-6. After 1 h, cells were treated with either DMSO (negative control, no inhibition) or 50 µM nirmatrelvir (positive control, full inhibition). Luminescence was measured at 30 h post-transfection. A minimum of 32 replicate wells were used for each control condition.

### 2.6. Evaluation of the Antiviral Activities Against SARS-CoV-2 Viruses

Experiments with live SARS-CoV-2 virus were performed in BSL-3 containment laboratories. The study utilized the hCoV-19/Russia/Moscow171619-031221/2021 strain (EPI_ISL_8920444, B.1.1.529 lineage) from the State Collection of Pathogens of Viral Infections and Rickettsioses at SRC VB “Vector” of Rospotrebnadzor (Russian Federation). Virus stocks were propagated in Vero E6 cells. For antiviral assays, Vero E6 cells were seeded in 96-well plates and grown to at least 95% confluence. The test compound, nirmatrelvir, was dissolved in dimethyl sulfoxide (DMSO) to a stock concentration of 10 mg/mL. The half-maximal inhibitory concentration (IC_50_) was determined using a cytopathic effect (CPE) reduction assay. Serial three-fold dilutions of nirmatrelvir were prepared starting from 600 μg/mL. Virus doses of 100 or 10 TCID_50_ per well were used. Cytotoxicity and antiviral activity were assessed simultaneously: compound dilutions were added to cell monolayers, followed by either plain medium (for cytotoxicity) or virus-containing medium (for antiviral activity). Plates were incubated at 37 °C for 4 days and then stained using the MTT assay. Absorbance was measured with a Thermo Scientific Multiskan FC plate reader, and data were analyzed in GraphPad Prism 8.0.0 (GraphPad Software, San Diego, CA, USA) using a four-parameter logistic curve. The 50% cytotoxic concentration (CC_50_), 50% inhibitory concentration (IC_50_), and selectivity index (SI = CC_50_/IC_50_) were calculated.

### 2.7. Assessment of Inhibitor Activity on a Fluorescent Substrate

The main SARS-CoV-2 protease used in this experiment was obtained as described previously. It was produced using a standard bacterial expression protocol [20] involving induction with IPTG. The purification of 3CLpro consisted of cell lysis by sonication, followed by clarification of the lysate and purification on Ni-Sepharose. The purity of the resulting 3CLpro sample was assessed by SDS-PAGE under denaturing conditions (Laemmli method), and the protein concentration was determined by the Bradford method [21].

To assess the ability of compounds to inhibit the SARS-CoV-2 main protease, IC_50_ values were determined. IC_50_ is defined as the half-maximal inhibitory concentration at which fluorescence decreases by 50% compared to the inhibitor-free control. Fluorescence arises from cleavage of the peptide substrate Dabcyl-KTSAVLQ↓SGFRKME(Edans)NH_2_ (CPC Scientific Inc., Rocklin, CA, USA) by 3CLpro. Fluorescence was recorded on a SuperMax 3100 fluorimeter (Flash, Shanghai, China) at excitation/emission wavelengths of 355/460 nm, respectively, in kinetic scanning mode.

Reaction mixtures containing Tris-HCl buffer (supplemented with EDTA, NaCl, DTT; pH 7.3), 3CLpro SARS-CoV-2 (1200 nM), and the test compound (0–200 µM concentration range) were incubated for 30 min in a 384-well plate at 30 °C, after which the reaction was initiated by adding the fluorogenic substrate (10 µM). IC_50_ values were calculated using a four-parameter logistic function.

### 2.8. Statistical Analysis

All statistical analyses were performed using GraphPad Prism 8.0.0 (GraphPad Software, San Diego, CA, USA). Data are presented as mean ± standard deviation (SD) from at least three independent biological replicates (*n* ≥ 3), each comprising three technical replicates per condition.

For pairwise comparisons between two groups, the nonparametric Mann–Whitney U test for independent samples was applied. For comparisons involving three or more groups, the Kruskal–Wallis test followed by Dunn’s post hoc test for multiple comparisons was used. Nonparametric tests were chosen because the distribution of luciferase signals in some experiments did not meet normality assumptions (Shapiro–Wilk test, *p* < 0.05 in some groups).

Differences were considered statistically significant at *p* < 0.05. Significance levels are indicated in the figures as follows: * *p* < 0.05; ** *p* < 0.01; *** *p* < 0.001; **** *p* < 0.0001; ns—not significant (*p* ≥ 0.05).

For IC_50_ determination, dose–response data were normalized to vehicle control (0% inhibition) and saturating inhibitor control (100% inhibition). IC_50_ values were calculated by nonlinear regression using a four-parameter logistic (4PL) model: Y = Bottom + (Top − Bottom)/(1 + 10^((LogIC_50_ − X) × HillSlope)), where Y is the normalized luminescence signal (% inhibition), X is the logarithm of inhibitor concentration, Bottom is the signal at maximal inhibition, and Top is the signal in the absence of inhibitor. The least squares fit was applied without constraining the parameters (unconstrained fit). IC_50_ values are reported as mean ± SD from at least three independent biological replicates, with 95% confidence intervals (95% CI) calculated where applicable.

## 3. Results

### 3.1. Selection of the Insertion Site in Luc for the SARS-CoV-2 3CLpro Cleavage Site

It is known that the C-terminal fragment of *Photinus pyralis* luciferase is critically important for the enzyme’s activity. Removal of the final 12 amino acid residues nearly abolishes detectable luminescence [22]. In this study, three potential surface-exposed sites were selected in the C-terminal region of Luc to ensure steric accessibility for interaction with 3CLpro. Positions G490, T508, and D520 were chosen based on an analysis of the Luc crystal structure (PDB ID: 1LCI). The selection criteria included surface location, the presence of an amino acid sequence of at least five residues, and proximity to the C-terminus. The VLQSGF sequence corresponding to the 3CLpro cleavage site was inserted at these sites.

**Figure 1:**
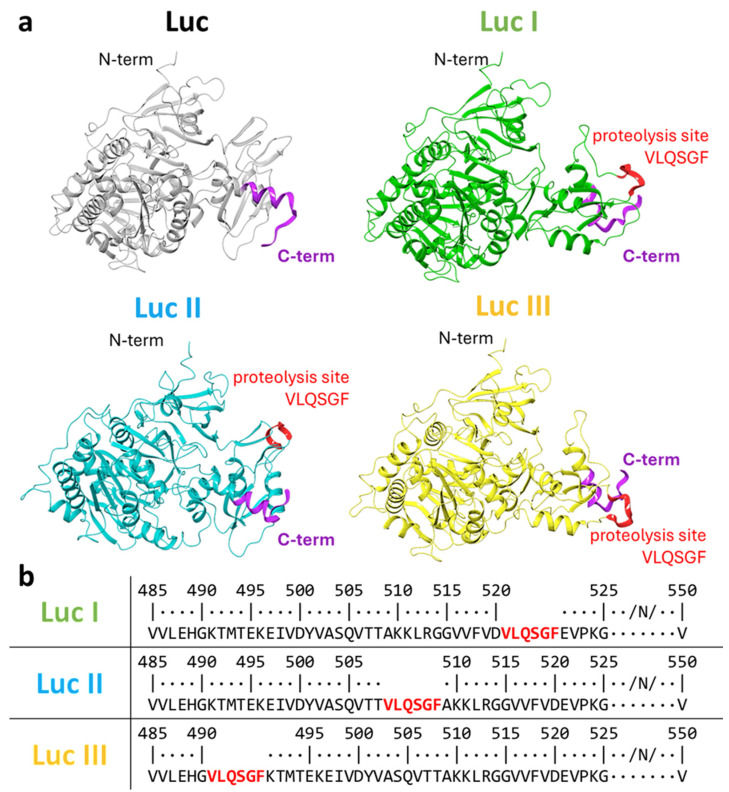
Modeling of luciferase variant structures. (**a**) Three-dimensional models of native firefly luciferase (Luc) and the three variants (Luc I, II, and III) with the inserted 3CLpro cleavage site VLQSGF. (**b**) Amino acid sequence alignment of the C-terminal region of native luciferase and the Luc I, II, and III variants. The positions of the VLQSGF site insertion are highlighted in color.

To theoretically assess the impact of the heterologous insertion, the structures of the engineered variants (Luc I, II, and III) were predicted using the AlphaFold3 methodology [23]. The structure obtained in a previous study [24] was employed for modeling (Figure 1a).

According to the pLDDT values, the luciferase structures consist of two domains: a large N-terminal domain and a smaller C-terminal domain (Figure S1). For the large domain, pLDDT values exceeded 90% for all four proteins (native Luc, Luc I, II, and III), indicating high prediction accuracy. In contrast, the structure of the small domain in variants II and III deviated noticeably from that of the reference structure. All predicted structures contained loop regions with confidence scores below 50%, suggesting that these parts of the protein are highly flexible. Notably, the terminal sequence of the C-terminal domain in variant III was predicted with the lowest confidence, which may indicate its potential mobility. Despite these local structural uncertainties, all variants retained the overall fold of the native enzyme (pLDDT > 90 for most residues; Figure S1 and Figure 1a), suggesting that catalytic activity was preserved.

### 3.2. Assessment of Luc Variant Activity Containing the Cleavage Site

**Figure 2:**
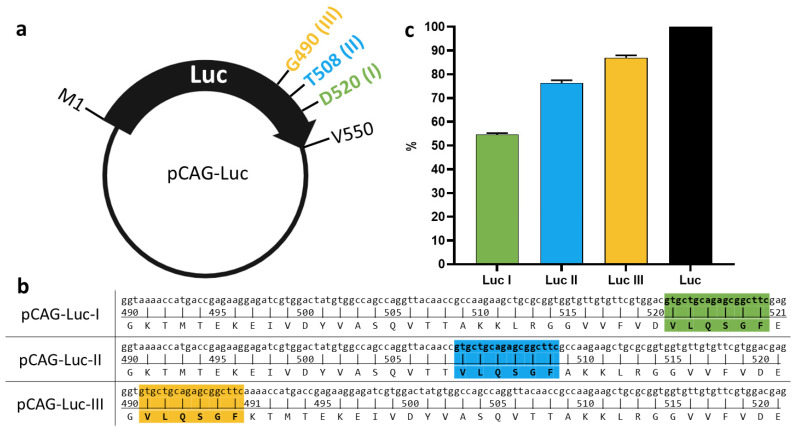
Activity of recombinant luciferase variants. (**a**) Schematic representation of the pCAG-Luc plasmid. G490 (III), T508 (II), and D520 (I) denote the amino acid residues after which the 3CLpro cleavage site (VLQSGF) is located. (**b**) Nucleotide and amino acid sequences of the region in plasmids pCAG-Luc-I, -II, -III encoding the luciferase gene. The location and sequence of the 3CLpro cleavage site (VLQSGF) are indicated in green, blue, and yellow. (**c**) Relative luminescence signal intensity (%). The luminescence signal of the pCAG-Luc control plasmid was set as 100%. Data are shown as geometric means ± SD. Statistical analysis was performed using the Mann–Whitney U test and the Kruskal–Wallis test.

To experimentally verify the retention of the luciferase variant properties after the insertion, plasmids pCAG-Luc-I, pCAG-Luc-II, and pCAG-Luc-III—encoding the Luc I, II, and III variants, respectively—were constructed. HEK293T cells were transfected with these plasmids, using HEK293T cells transfected with pCAG-Luc as a positive control. All three variants retained enzymatic activity, though activity levels varied significantly (Figure 2c). Compared to native Luc, the activity of Luc I decreased by 45%, that of Luc II by 22%, and that of Luc III by only 12%.

**Figure 3:**
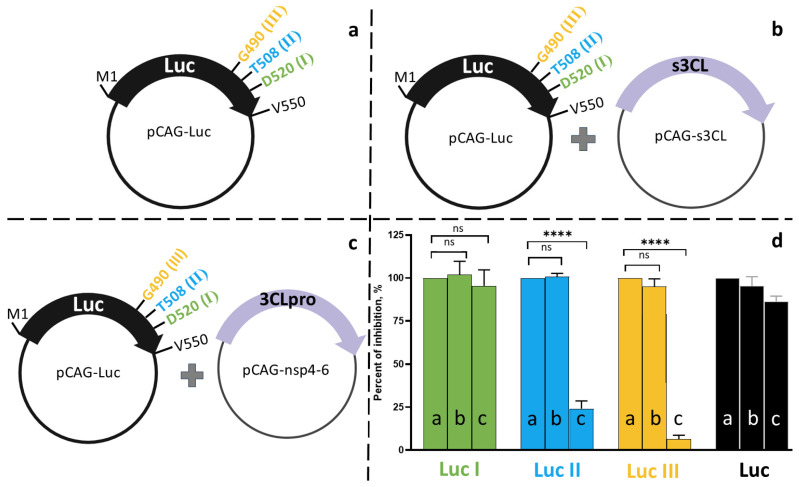
Effect of 3CLpro co-transfection on luminescence levels in HEK293T cell lysates 24 h post-transfection. (**a**) Transfection of HEK293T cells with a single plasmid carrying the luciferase gene: pCAG-Luc (black bar), pCAG-Luc-I, pCAG-Luc-II, pCAG-Luc-III (green, blue, and yellow bars, respectively). (**b**) Co-transfection of HEK293T cells with a luciferase gene-containing plasmid and the pCAG-s3CL plasmid encoding the mature 3CL protease. (**c**) Co-transfection of HEK293T cells with a luciferase gene-containing plasmid and the pCAG-nsp4-6 plasmid encoding the SARS-CoV-2 polyprotein fragment (nsp4-nsp6). (**d**) Relative luminescence signal (%). For each luciferase variant (Luc I, II, III), the luminescence signal obtained upon transfection with the luciferase-encoding plasmid alone (without any protease) was set as 100% (indicated by the dashed line). Bars show the residual luminescence upon co-transfection with pCAG-s3CL (**b**) or pCAG-nsp4-6 (**c**). Data are shown as mean ± SD (*n* = 3 independent experiments, each with three technical replicates). Individual data points are overlaid on the bars. Unpaired *t*-test (**** *p* < 0.0001; ns, not significant, *p* > 0.5).

To assess the functionality of the embedded protease site, we compared the effects of two protease expression variants: the mature 3CLpro form (Figure 3b) and the more extended polyprotein fragment nsp4-nsp6, which better mimics the native processing context (Figure 3c). The Luc I construct showed no response to protease presence in either form—luminescence levels remained unchanged upon co-transfection with either the plasmid encoding the mature 3CLpro or with pCAG-nsp4-6. In contrast, the Luc II and Luc III variants exhibited significant reductions in luciferase activity (by 75% and 90%, respectively), but exclusively upon co-transfection with pCAG-nsp4-6 (Figure 3d, columns c). Co-expression of Luc II and Luc III with the mature 3CLpro form did not alter the signal (Figure 3d, columns b).

**Figure 4:**
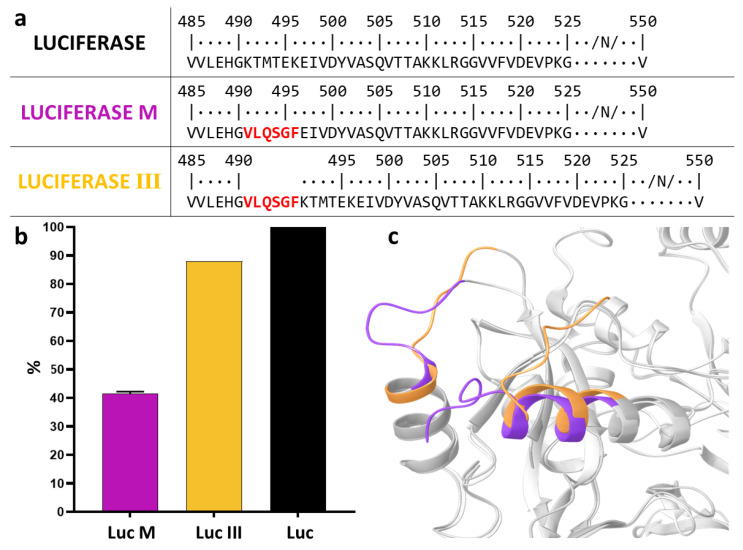
Effect of the (VLQSGF) loop on luciferase functional activity. (**a**) Amino acid sequence segment of luciferase variants (Luc M and Luc III) compared to the native form (Luc). (**b**) Relative luminescence signal (%). HEK293T cells were transfected with pCAG-Luc (native luciferase, control), pCAG-Luc-M (full loop replacement), or pCAG-Luc-III (insertion after G490). Luciferase activity was measured 24 h post-transfection. The luminescence signal obtained with pCAG-Luc (native luciferase) was set as 100%. Data are shown as mean ± SD (*n* = 3 independent experiments). (**c**) Computational modeling of the loop region of the mutant luciferase variants compared to the native form.

Further development of the cell-based system focused on the Luc III variant, which exhibited the largest difference in luminescence signal upon co-transfection. According to computational modeling (Figure 4c), the insertion of the additional six amino acids in Luc III resulted in an expansion of the loop within the helix-loop-helix motif and a minor change in luciferase activity. To confirm that the activity reduction in Luc III upon co-transfection was due to cleavage specifically at the inserted site rather than to conformational changes, we replaced the entire loop (helix-loop-helix) with the VLQSGF site, creating the Luc M variant (Figure 4a). Transfection of HEK293 cells with the pCAG-Luc-M plasmid encoding this variant led to a greater drop in activity (Figure 4b). This underscores the importance of the native structure of this region for enzyme function and confirms that the moderate activity reduction in Luc III results from a more conservative insertion.

When using a co-transfection strategy with two independent plasmids (pCAG-Luc-III and pCAG-nsp4-6), the system’s efficiency is limited by two factors. First, the delivery of both plasmids into the same cell is not 100% efficient, leading to population heterogeneity and reduced signal-to-noise ratio. Second, the simultaneous expression of two foreign proteins under the control of strong promoters imposes a substantial metabolic burden on the host cell [25]. Competition for limited cellular resources (ribosomes, elongation factors, ATP) can affect the expression levels of both genes and, consequently, the reproducibility of the results.

**Figure 5:**
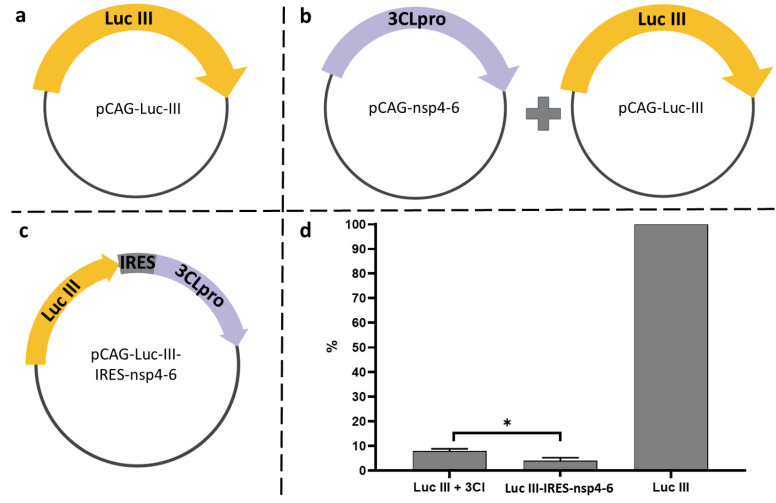
Comparison of co-transfection and bicistronic expression schemes. (**a**) Co-transfection with plasmids pCAG-Luc-III and pCAG-nsp4-6; (**b**) transfection with the bicistronic plasmid pCAG-Luc-III-IRES-nsp4-6; (**c**) control transfection with pCAG-Luc-III. (**d**) Relative luciferase activity (%). The luminescence signal from control cells transfected with pCAG-Luc-III alone (**c**) was set as 100%. All other conditions are expressed relative to this control. Data are shown as mean ± SD (*n* = 3 independent experiments). Unpaired *t*-test (* *p* < 0.05).

To address these limitations and enhance the system’s efficiency, a bicistronic plasmid, pCAG-Luc-III-IRES-nsp4-6, was constructed (Figure 5c). In this construct, the Luc-III and nsp4-6 genes are arranged within a single transcriptional unit under the control of a common CAG promoter. The separate translation of the proteins is enabled by the internal ribosome entry site (IRES) from the encephalomyocarditis virus. This design ensures that any cell expressing the Luc-III reporter gene will also express the viral protease.

A comparative analysis revealed that the bicistronic construct pCAG-Luc-III-IRES-nsp4-6 produced an 8% greater reduction in luciferase activity compared to co-transfection (Figure 5d). This confirms that the bicistronic system provides more efficient and coordinated expression of both elements, minimizing the variability associated with co-transfection and enhancing the sensitivity of inhibitor detection.

### 3.3. Validation of the System Using 3CLpro Inhibitors

For the functional validation of the developed cell-based system and to assess its suitability for screening potential 3CLpro inhibitors, a panel of four compounds with known activity against the main coronavirus protease was tested. The panel included nirmatrelvir (PF-07321332)—a clinically approved 3CLpro inhibitor [26], GC376—a broad-spectrum inhibitor of viral proteases from the 3C and 3CL families [10,27], ML188—the first described non-covalent inhibitor of SARS-CoV-1 3CLpro [28], and disulfiram—a drug for treating alcohol dependence, considered as a potential 3CLpro inhibitor [5].

To validate the system, these four compounds were tested using the following procedure. HEK293T cells transfected with the bicistronic plasmid pCAG-Luc-III-IRES-nsp4-6 were treated with test compounds 1 h post-transfection, and luciferase activity was measured after 30 h. Non-specific effects on luciferase were assessed using cells transfected with pCAG-Luc-III alone. The cytotoxicity (CC_50_) of the compounds was evaluated in parallel using the standard MTT assay. For comparative analysis, all experiments included a cell-free FRET assay with the recombinant 3CLpro enzyme, and measurements using the live SARS-CoV-2 virus model (Wuhan variant) in Vero E6 cell culture.

**Figure 6:**
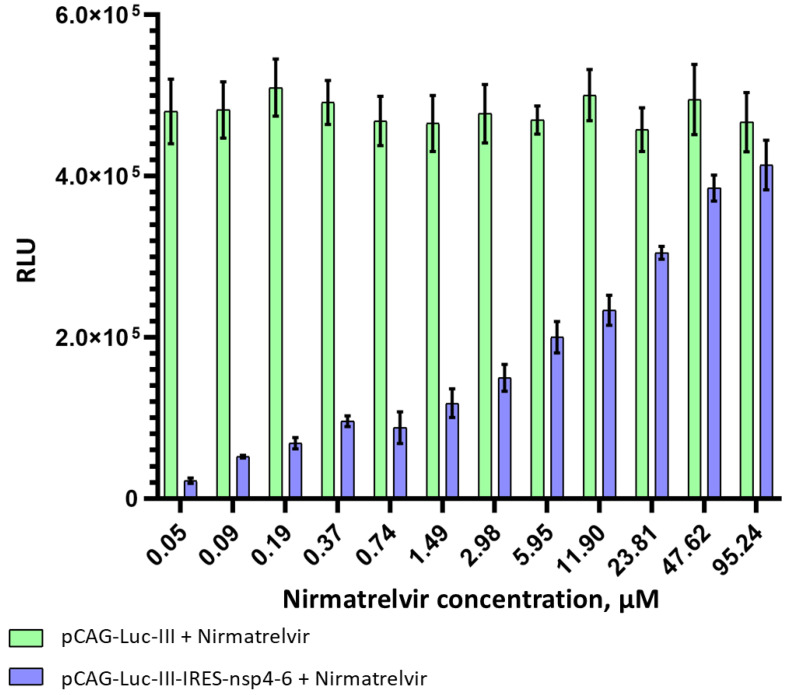
Dependence of luciferase activity on nirmatrelvir concentration. HEK293T cells were transfected with pCAG-Luc-III (control, no protease; green) or pCAG-Luc-III-IRES-nsp4-6 (expressing both luciferase reporter and viral protease; blue). Nirmatrelvir was added 1 h post-transfection at the indicated concentrations. Luciferase activity was measured 30 h post-transfection. Data are shown as raw luminescence values (relative light units, RLU). Data are presented as mean ± SD (*n* = 3 independent experiments).

None of the tested compounds, in the concentration range studied, had a significant effect on luciferase activity in control cells transfected only with pCAG-Luc-III, confirming the absence of non-specific effects of the compounds on the reporter protein. In Figure 6, this result is shown using nirmatrelvir as an example (green bars). In cells expressing the viral protease (pCAG-Luc-III-IRES-nsp4-6), all compounds showed a classic dose–response relationship: minimal luminescence levels at low inhibitor concentrations and an increase in signal with increasing concentrations. For nirmatrelvir, this relationship is presented in Figure 6 (blue bars).

The luciferase activity of the lysates was converted to percent inhibition, and the obtained data were approximated using a four-parameter logistic model (4PL) in GraphPad Prism 8. The calculated IC_50_ values for all tested compounds in the developed luciferase system, as well as comparative data from the FRET analysis and the viral tests, are presented in Table 1. For nirmatrelvir, the IC_50_ value in the developed system was 6.1 ± 0.8 µM, which is comparable to the value obtained in the viral tests (3.6 ± 0.5 µM), while in the FRET analysis, this value was significantly lower (0.105 ± 0.009 µM). For GC376, the IC_50_ value in the luciferase system was 3.3 ± 1.1 µM versus 0.023 ± 0.004 µM in the FRET analysis and 9.54 ± 2.03 µM in viral tests. ML188 showed an IC_50_ of 1.56 ± 0.55 µM in the FRET analysis and 3.77 ± 0.87 µM in the viral tests; however, due to its relatively high toxicity to HEK293T cells, its inhibitory effect in our system could not be reliably measured.

Disulfiram showed no inhibitory activity in the developed cell system, consistent with its lack of effect on the live virus. Similarly, the drug Remdesivir, which is an inhibitor of RNA-dependent RNA polymerase and blocks SARS-CoV-2 in cell culture, showed no activity in the developed system. All tested compounds, except ML188, demonstrated low cytotoxicity to HEK293T cells (CC50 > 90–100 µM), confirming that the observed decrease in luciferase activity was not associated with cell death.

To assess the suitability of the system for high-throughput screening, we calculated the Z′-factor, signal-to-background (S/B) ratio, and coefficient of variation (CV) using 32 replicate wells of negative (DMSO) and positive (50 µM nirmatrelvir) controls. The Z′-factor was 0.67, the S/B ratio was 8.4, and the CV was 8.3%. These values meet or exceed standard acceptance criteria for HTS assays (Z′ > 0.5, CV < 20%), confirming the robustness of the system.

## 4. Discussion

### 4.1. General Context and Rationale

The COVID-19 pandemic has once again demonstrated that the development of high-throughput assays is of great importance for discovering antiviral drugs and for rapidly responding to viral outbreaks. Viral proteases remain attractive targets to this day due to their decisive role in viral polyprotein processing. Currently, numerous inhibitors of HIV-1 protease and hepatitis C virus NS3/4A protease are used in clinical practice (e.g., amprenavir, tipranavir for HIV-1; asunaprevir, paritaprevir for hepatitis C virus).

In this work, we developed and validated a new cell-based system for screening viral protease inhibitors, using the main protease of the SARS-CoV-2 coronavirus as a model. The system is based on the split luciferase complementation of *Photinus pyralis* luciferase, with the proteolysis site embedded directly in the C-terminal region of the enzyme. This design allows for the tracking of protease activity by a decrease in luminescence upon cleavage of the luciferase molecule, and the monitoring of its inhibition by signal recovery.

A critical consideration in developing any cell-based assay is the choice of the cellular model. For a transfection-based screening system, HEK293T cells offer practical advantages, including high transfection efficiency (>95%) and the absence of endogenous proteases recognizing the VLQSGF site. While differentiated respiratory epithelial cells such as Calu-3 are more physiologically relevant for SARS-CoV-2 infection, their low transfectability makes them unsuitable for this platform. Importantly, IC_50_ values obtained in HEK293T correlated well with live virus data on Vero E6 cells (Table 1).

### 4.2. Selection of the Optimal Luciferase Variant

Previous studies have shown that the C-terminal region of firefly luciferase is critically important for enzymatic activity: the removal of just 12 amino acids leads to an almost complete loss of function [29]. We utilized this phenomenon by inserting the 3CLpro recognition site (VLQSGF) into three different surface regions of the protein near the C-terminus (G490, T508, and D520), selected based on the crystal structure of Luc (PDB ID: 1LCI). However, this experimentally determined structure has notable limitations: it contains gaps in the amino acid sequence and, crucially, lacks structural information for the last eight C-terminal residues. These gaps complicate rational design, as the precise conformation and accessibility of the C-terminal region—critical for both enzyme activity and insertion of a protease cleavage site—cannot be reliably inferred from the available crystallographic data alone. Therefore, to overcome this limitation and obtain complete three-dimensional models of the engineered variants, we turned to computational prediction using AlphaFold3.

Both modeling and experimental data confirmed that all three variants retained enzymatic activity, albeit at different levels. Compared to native Luc, activity was reduced by 45% for Luc I (insertion after D520), 22% for Luc II (insertion after T508), and only 12% for Luc III (insertion after G490). The difference in activity reduction likely reflects the varying degrees of structural disruption caused by the insertions at different positions in the C-terminal region. The minimal impact on Luc III activity suggests that the region around G490 can accommodate a six-amino-acid insertion with relatively minor conformational changes, making this variant the most suitable candidate for biosensor development.

However, upon comparing the predicted structures, we observed a paradox: according to the modeling, the fold of the C-terminal domain in variant III differed significantly from that observed in the PDB structure of native luciferase. Nevertheless, variant III retained the highest activity (88% of the native level). This discrepancy between theoretical prediction and experimental data suggests that, in this case, AlphaFold may not have accurately captured the relevant structural features. The most likely explanation is the high mobility of the C-terminal region, particularly the terminal sequence of variant III, as supported by the low pLDDT values for this region. Such mobility may result in the in silico prediction failing to reflect the actual conformational dynamics of the protein in solution. Consequently, the insertion of the cleavage site at position G490 appears not to disrupt the functionally relevant structure as severely as the modeling might suggest.

### 4.3. Importance of the Native Polyprotein Context for Protease Activity

A key finding of our study is that the mature, isolated form of 3CLpro (s3CL) failed to cleave the luciferase reporter, whereas the extended polyprotein fragment nsp4–nsp6 efficiently did so. This observation highlights a critical aspect of coronavirus protease biology that is often overlooked in conventional cell-free or cell-based assays using recombinant mature proteases.

Several non-mutually exclusive mechanisms may explain this requirement for the polyprotein context. First, the nsp4–nsp6 fragment includes the natural N-terminal flanking region of 3CLpro, which contains a hydrophobic sequence that may act as a signal peptide or membrane anchor. This sequence could facilitate the association of the protease with intracellular membranes, particularly the endoplasmic reticulum, where coronavirus replication complexes are assembled. Such membrane association may concentrate the protease in close proximity to its natural substrates (including nsp4–nsp6 itself and the luciferase reporter) and create a favorable microenvironment for proteolysis. Second, the polyprotein context may be essential for the proper folding of 3CLpro. It is well established that many viral proteases undergo co-translational folding and require their flanking regions to achieve the correct three-dimensional structure. Expression of mature 3CLpro in isolation may lead to misfolding or aggregation, particularly in the reducing environment of the cytoplasm. Third, the nsp4–nsp6 fragment may be necessary for the autoprocessing activity of 3CLpro. The release of mature 3CLpro from the viral polyprotein occurs through cis- and trans-cleavage events that require the protease to be embedded within the polyprotein. Expressing 3CLpro in its mature form bypasses these autoprocessing steps, potentially resulting in an enzyme that is not fully activated. Fourth, the flanking regions may stabilize the protease or protect it from cellular degradation pathways, thereby increasing its steady-state concentration and half-life.

Our findings are consistent with previous observations for other coronaviruses. For example, Kilianski et al. demonstrated that a polyprotein context was also required for the activity of MERS-CoV 3CLpro and PLpro in luciferase-based biosensors [17]. Similarly, the processing of the SARS-CoV-2 replicase polyprotein is known to occur within membrane-associated replication complexes, where the spatial organization of viral proteins plays a critical role. Taken together, these observations suggest that assays relying on isolated mature proteases may not fully capture the activity of these enzymes in the absence of a cellular or polyprotein context. By preserving the native polyprotein arrangement, our system offers a more representative platform for inhibitor screening compared to assays using purified proteases alone.

### 4.4. Bicistronic Expression Improves Reproducibility

Our initial experiments using co-transfection with two separate plasmids (pCAG-Luc-III and pCAG-nsp4-6) demonstrated the validity of the approach but revealed potential limitations. The efficiency of co-transfection in mammalian cells rarely reaches 100%, leading to a heterogeneous cell population where some cells express only the reporter, only the protease, or both proteins. This heterogeneity reduces the signal-to-noise ratio and introduces variability. Additionally, the simultaneous expression of two foreign proteins under the control of strong promoters creates a metabolic burden on the host cells [25], potentially affecting cell viability and the reproducibility of the results. To address these issues, we constructed a bicistronic plasmid (pCAG-Luc-III-IRES-nsp4-6), in which both genes are transcribed as a single mRNA but translated separately via the encephalomyocarditis virus IRES element. This design ensures that any cell expressing the reporter gene Luc-III also expresses the viral protease, eliminating the heterogeneity inherent in co-transfection systems. The bicistronic configuration resulted in an 8% greater reduction in luminescence compared to co-transfection, indicating more efficient and coordinated expression of both components. This improvement aligns with the expected stoichiometric ratio between the reporter and the protease in the bicistronic system, whereas co-transfection can result in variable plasmid ratios entering the cells.

### 4.5. Validation of the System with Known 3CLpro Inhibitors

To validate the functionality and specificity of our biosensor system, we tested a panel of compounds with known activity against 3CLpro. Nirmatrelvir, an orally available 3CLpro inhibitor that received emergency use authorization for COVID-19 treatment [26], served as the positive control. The calculated IC_50_ values for all tested compounds in the developed luciferase system, as well as the comparative data from the FRET analysis and the viral assays, are presented in Table 1. For nirmatrelvir, the IC_50_ value in the developed system was 6.1 ± 0.8 µM, which is comparable to the value obtained in the viral assays (3.6 ± 0.5 µM), whereas in the FRET analysis, this value was significantly lower (0.105 ± 0.009 µM). This discrepancy between cell-free and cell-based assays is often observed and reflects the additional barriers that compounds must overcome in the cellular environment, including membrane permeability, efflux pump activity, protein binding, and metabolic stability [6,30].

The close correspondence between our biosensor and the live virus data suggests that the biosensor faithfully reproduces the cellular context in which viral protease inhibition occurs. GC376, a broad-spectrum inhibitor of the 3C and 3CL protease families, initially developed for veterinary use [31,32], showed similar behavior with an IC_50_ of 3.3 ± 1.1 µM in our system compared to 0.023 ± 0.004 µM in the FRET analysis and 9.54 ± 2.03 µM in the viral assays.

ML188, discovered through the high-throughput screening of non-covalent inhibitors of the SARS-CoV-1 main protease [33], showed an IC_50_ of 1.56 ± 0.55 µM in the FRET analysis and 3.77 ± 0.87 µM in the viral assays. However, it exhibited relatively high toxicity to HEK293T cells (CC_50_ 90 ± 4 µM), and as a result, its inhibitory effect in our system could not be reliably measured. This result highlights an important advantage of cell-based assays over biochemical screenings: they allow for the identification of compounds that, while active in vitro, cannot act on their target in a cellular context due to various biological barriers. Disulfiram, which was considered a potential 3CLpro inhibitor based on computational studies and preliminary biochemical data [34] showed no inhibitory activity in the developed cellular system, consistent with its lack of effect on the live virus. These results are consistent with data from other researchers [35] and suggest that disulfiram is unlikely to be a useful 3CLpro inhibitor in a therapeutic context, underscoring the importance of experimental validation that goes beyond computational predictions and simple biochemical tests. As an additional control for specificity, we tested remdesivir, an RNA-dependent RNA polymerase inhibitor active against SARS-CoV-2 [36]. As expected, remdesivir showed no inhibition in our biosensor system, despite its high activity against the live virus (IC_50_ = 3.76 ± 0.94 µM), confirming that the observed signal recovery is specific to 3CLpro inhibition and not associated with general antiviral effects or non-specific cytotoxicity. None of the tested compounds had any significant effect on luminescence in cells transfected with the control plasmid pCAG-Luc-III (without the protease), confirming the absence of non-specific effects on luciferase activity or cell viability at the concentrations studied. Cytotoxicity tests (MTT) confirmed that all tested compounds, except ML188, have low cytotoxicity to HEK293T cells (CC_50_ > 90–100 µM).

### 4.6. Comparison with Existing Luciferase-Based Biosensors

Luciferase biosensors for viral proteases reported in the literature can be divided into two main types: those based on circularly permuted Luc variants [15,16,17,18] and those based on NanoLuc complementation [19]. The first type requires complex protein design, while the second requires the use of an expensive substrate. The system we develop is free from these limitations. It is based on the simple insertion of the cleavage site into the structure of native luciferase, allowing for the use of standard luciferin. This design provides high basal activity (up to 88% of wild type) and a more than 90% signal reduction after cleavage. The bicistronic expression format further enhances reproducibility and ease of use, ensuring coordinated expression of all components from a single plasmid.

The strong correlation between the IC_50_ values obtained with our biosensor system and those from live virus assays suggests that this platform can serve as a reliable surrogate for virus-based screening, with the potential for high-throughput use in BSL-2 laboratories without requiring BSL-3 conditions. This is particularly valuable for emerging viruses where working with the live virus is dangerous or requires specialized facilities. The system can be adapted for other viral proteases by simply replacing the cleavage site sequence and, if necessary, the polyprotein context. For proteases requiring cofactors or having specific membrane association requirements, the bicistronic construct can be modified to include these elements.

### 4.7. Limitations of the Study

Despite the advantages described above, several limitations of the present study should be acknowledged. First, the system was optimized for the HEK293T cell line, which is not physiologically relevant for respiratory viruses such as SARS-CoV-2. Results may differ in cells with different metabolic activity, expression profiles of efflux pumps (e.g., P-glycoprotein), or intracellular protease repertoires. Second, the use of the strong constitutive CAG promoter leads to supraphysiological expression levels of both the luciferase reporter and the viral protease. While this produces a robust and easily detectable signal, it may create an artificially high protease “load” that could reduce the sensitivity of the assay to weak inhibitors, potentially resulting in higher IC_50_ values compared to viral infection assays (as shown in Table 1 for nirmatrelvir and GC376). Third, unlike a complete viral infection, our system does not include other viral proteins (e.g., PLpro, RdRp), which could influence the intracellular availability of inhibitors or serve as alternative targets for the tested compounds. Fourth, the artificial insertion of the 3CLpro cleavage site into the luciferase structure, although minimized by selecting position G490 (Luc III), may still affect enzyme folding to some extent. Fifth, the system cannot evaluate inhibitors that act on other stages of the viral life cycle, such as viral entry, RNA replication, or virion assembly. Finally, validation in other cell types relevant to viral infection (e.g., respiratory epithelial cells such as Calu-3 or primary bronchial epithelial cells) remains an important task for future research. These limitations should be considered when interpreting the results and planning subsequent studies.

### 4.8. Future Perspectives

The developed platform can be directly adapted for testing inhibitors against mutant variants of 3CLpro. To do this, it is sufficient to replace the nsp4-6 fragment in the bicistronic construct pCAG-Luc-III-IRES-nsp4-6 with the corresponding mutant gene containing the amino acid substitutions of interest (e.g., E166V, L167F, S144A, etc.). The luciferase part remains unchanged because the VLQSGF cleavage site is preserved in most cases (unless the mutation affects the P-positions of the recognition site).

It is expected that for mutants with known resistance to inhibitors (e.g., E166V to nirmatrelvir), the IC_50_ values in our system will be increased proportionally to those in viral assays. However, as noted in Section 4.7, due to protease overexpression under the control of a strong CAG promoter, the absolute IC_50_ values in our system may be systematically higher than those during viral infection. Nevertheless, the relative resistance (fold change in IC_50_ of mutant versus wild-type) should correlate with data obtained from live virus. This allows our platform to be used for rapid assessment of the risk of resistance development to new inhibitors without the need to work with live virus in BSL-3 conditions. Direct comparison of results for isogenic 3CLpro mutants is the subject of our ongoing research.

Moreover, we are adapting the developed platform to create a similar biosensor system targeting HIV-1 protease. Considering that HIV-1 protease functions as a homodimer and recognizes specific sequences in the Gag and Gag-Pol polyproteins, we are modifying the construct design by inserting the corresponding proteolysis site (e.g., the SQNYPIV sequence) into the C-terminal region of the Luc III variant. To provide the proper dimerization context and to allow for autoprocessing, we plan to use a bi-cistronic system including an HIV-1 polyprotein fragment, similar to the successful strategy applied for SARS-CoV-2 3CLpro. This will enable the making of an adaptable platform for screening HIV-1 protease inhibitors under conditions as close as possible to the native ones, expanding the scope of our platform’s application to various viral targets.

## 5. Conclusions

In this study, we developed a simple, reliable, and cost-effective cell-based biosensor system for screening viral protease inhibitors, using SARS-CoV-2 3CLpro as a proof-of-concept. The system correctly identified clinically relevant inhibitors (nirmatrelvir and GC376), distinguished them from inactive compounds (disulfiram), and showed a strong correlation with live virus data. The inclusion of the native polyprotein context (nsp4-nsp6) proved essential for proper protease function, emphasizing the importance of recreating physiological conditions in the development of cell-based assays. The bicistronic expression format ensures the coordinated expression of all components and improves reproducibility. This platform can be readily adapted for other viral proteases and has the potential to accelerate the discovery and development of new antiviral drugs.

## Figures and Tables

**Table 1:** Comparison of Cytotoxicity (CC50) and Inhibitory Activity (IC50) of Compounds in Enzymatic, Cell-Based, and Virological Assays. Data are shown as mean ± SD from at least three independent experiments.

Compound	CC_50_, μM (HEK293T)	IC_50_, μM (Luc System)	IC_50_, μM (rec-3CLpro)	IC_50_, μM (Virus)
Nirmatrelvir	>100	6.1 ± 0.8	0.105 ± 0.009	3.6 ± 0.5
GC376	>100	3.3 ± 1.1	0.023 ± 0.004	9.54 ± 2.03
ML188	90 ± 4	NA	1.56 ± 0.55	3.77 ± 0.87
Disulfiram	101 ± 6	-	6.25 ± 1.97	-
Remdesivir	>100	-	-	3.76 ± 0.94

NA—measurement could not be performed.

## Data Availability

The original contributions presented in the study are included in the article, and further inquiries can be directed to the corresponding author.

## Supplementary Materials

The following supporting information can be downloaded at: https://www.mdpi.com/article/10.3390/bios16050253/s1, Figure S1: The result of predicting the tertiary structures of luciferases: the secondary structure of the protein is colored according to the pLDDT estimate.
