# Supplementary material for: Cell-Based Luciferase Assay for Testing SARS-CoV-2 3CL Protease Inhibitors
Source: Biosensors (Basel). 2026 Apr 30;16(5):253. doi: 10.3390/bios16050253 (PMC13204911; doi:10.3390/bios16050253)
Supplement: Supplementary file 1 [file biosensors-16-00253-s001.zip › biosensors-4249165-supplementary.pdf]

Article

Cell-Based Luciferase Assay for Testing SARS-CoV-2 3CL
Protease Inhibitors

Dmitry N. Shcherbakov [1,2], Ekaterina D. Mordvinova [1,3], Vadim O. Trufanov [1,3], Natalia V. Volkova [1], Yulia V. Meshkova [3], Maria K. Marenina [3], Anna V. Zaykovskaya [1], Ekaterina A. Volosnikova [1], Sophia S. Borisevich [4] and Svetlana V. Belenkaya [1,3,*]

1 State Research Center of Virology and Biotechnology VECTOR, Rospotrebnadzor, 630559 Novosibirsk, Russia; scherbakov_dn@vector.nsc.ru (D.N.S.); mordvinova97@mail.ru (E.D.M.); trufano8@mail.ru (V.O.T.); tasha_wolkowa11.93@mail.ru (N.V.V.); zaykovskaya_av@vector.nsc.ru (A.V.Z.); volosnikova_ea@vector.nsc.ru (E.A.V.)

2 Research Institute of Biological Medicine Center for Recombinant Technologies, Altay State University, 656049 Barnaul, Russia

3 N. N. Vorozhtsov Novosibirsk Institute of Organic Chemistry, Siberian Branch of the Russian Academy of Sciences, Academician Lavrent'ev Ave. 9, 630090 Novosibirsk, Russia; meshkova@nioch.nsc.ru (Y.V.M.); mareninamk@nioch.nsc.ru (M.K.M.)

4 Synchrotron Radiation Facility, Siberian Circular Photon Source "SKIF" Boreskov Institute Catalysis of
Siberian Branch of the Russian Academy of Sciences, Nikolskiy pr-t, 1, Koltsovo, 630559 Novosibirsk, Russia; monrel@mail.ru

* Correspondence: belenkaya.sveta@gmail.com

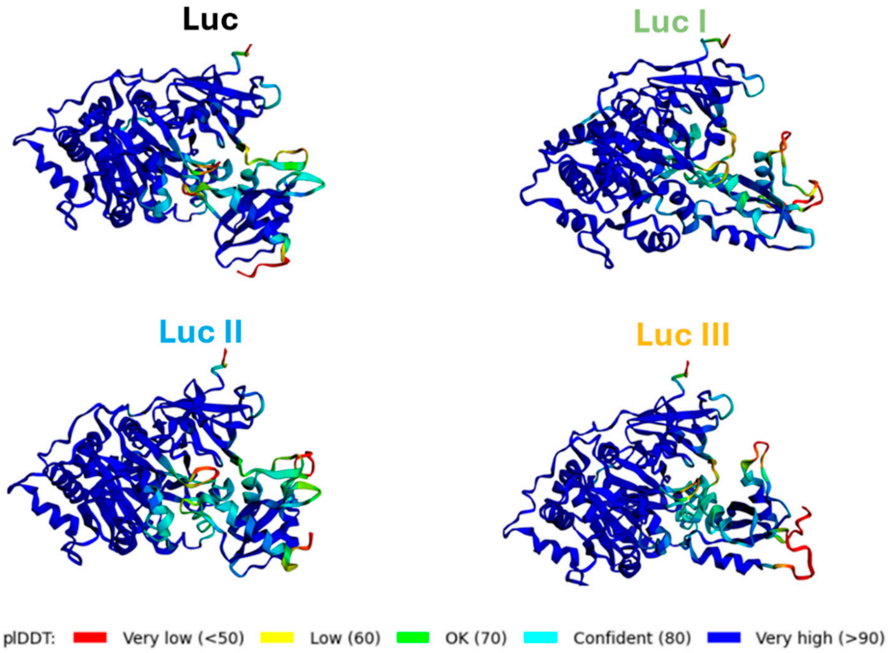

Figure S1. The result of predicting the tertiary structures of luciferases: the secondary structure of the protein is colored according to the pLDDT estimate.
